# Views and Experiences of Online Exercise Groups for People With Parkinson’s Disease: A Qualitative Study

**DOI:** 10.1155/padi/9816558

**Published:** 2026-01-28

**Authors:** James Alexander, Caroline Appel, Yiota Constantinou, Anette Schrag

**Affiliations:** ^1^ Department of Clinical and Movement Neurosciences, Queen Square Institute of Neurology, University College London, London, UK, ucl.ac.uk; ^2^ Camden Neurology & Stroke Service, Central & North West London NHS Foundation Trust, London, UK; ^3^ Department of Allied Health, School of Health and Medical Sciences, City St George’s University of London, London, UK

**Keywords:** digital health, e-health, exercise, group-based exercise, online exercise, Parkinson’s disease, physical activity, qualitative

## Abstract

**Background:**

Online exercise groups for people with Parkinson’s disease (PwP) are increasing in popularity, but little is known about PwP’s experiences with them.

**Objective:**

To explore the views and experiences of PwP who have utilised Parkinson’s disease‐specific online exercise groups.

**Methods:**

A qualitative study utilising semistructured interviews and thematic analysis in a purposive sample of PwP who had participated in an online exercise group.

**Results:**

Nine participants (5 females) with a mean age of 69.5 (63–78) years and a mean disease duration of 9.1 (3–20) years participated. Analysis revealed three overarching themes: *‘Considerations of online exercise groups for PwP*’, which highlighted the pros and cons of attending online exercise classes; ‘*Online exercise class qualities’*, including the importance of a tailored approach, clearly communicated aims and the importance of a well‐informed instructor; and ‘*Accessibility’*, which included considerations of convenience of access, costs and technological access.

**Conclusion:**

Online exercise groups may play an important role in future Parkinson’s disease management by offering greater access to exercise. They may also perpetuate inequalities for PwP and lack the social engagement many PwP seek. Hybrid group exercise, a combination of online and face‐to‐face classes, could provide this. Providers must develop classes that are tailored to PwP and delivered by well‐informed instructors.

## 1. Introduction

Parkinson’s disease (PD) is the second most prevalent neurodegenerative condition after Alzheimer’s disease [1]. Its key features include slowness, stiffness, tremor, postural instability, depression and other non‐motor symptoms [2]. Compared to the general population, people with PD (PwP) are typically one‐third less active [2]. This is attributed to reduced physical abilities (related to strength, balance and coordination), mental health impairments (low mood and apathy) and personal factors (including reduced self‐efficacy) [2–4].

One type of physical activity is exercise, describing movements of the body by skeletal muscles that require energy expenditure and are planned, structured and purposeful, aiming to maintain or improve at least one component of physical fitness [5]. Exercise is considered by some as important as medication for managing PD symptoms [6]. In PwP, it has shown improvements in gait, [7] strength, [8] balance, [9] mood and quality of life [10]. Hirsch et al. [11] have suggested neuroprotective and neurorestorative effects of high‐intensity exercise for PwP, and this continues to be an area for future research. Due to the heterogeneity of PD and individual characteristics, no one type of exercise is recommended over another, and exercise can be performed individually, in a group, supervised or unsupervised [2].

Group exercise remains popular for PwP due to its nurturing and supportive environments and social cohesion [12]. Group exercise such as dance, high‐intensity interval training (HIIT) and tai chi has shown health benefits for PwP [13–16]. However, those with transport or mobility issues or in isolated communities have limited access to in‐person groups [17, 18]. A need for greater equity of access to exercise has led to a need to explore remote forms of group exercise [19]. Studies exploring online group exercise for PwP have shown good feasibility, adherence and self‐reported health benefits [20–23]. Online exercise groups can reduce some of the known external barriers to participating in exercise including time, access, cost and transport, as well as offering a more flexible approach to exercise around daily lives [24].

Since the COVID‐19 pandemic, online exercise groups have remained popular with PwP and health and exercise professionals. Online exercise groups could potentially form a larger part of healthcare and community services by improving equity of access to exercise and addressing patient preferences. There is, however, a lack of information on the benefits of and experiences with online exercise groups by PwP and, therefore, a need to further explore and review this type of intervention [25]. There has been little research into PwP’s experiences of online exercise groups to support PwP to manage their health and inform the design of services to meet their needs [25]. This study explored experiences of PwP with online exercise groups and specifically their perceived value, impact and effectiveness.

## 2. Materials and Methods

The Standards for Reporting Qualitative Research checklist was used to report this study [26]. The study used an interpretivist design underpinned by phenomenological theory. Semistructured online interviews were conducted to explore the lived experiences of PwP who participated in online exercise groups [27]. A patient and public involvement group (PPI), comprising people living with Parkinson’s, was established with support from Parkinson’s UK. The PPI group supported the development of the research question and study methodology.

### 2.1. Ethics

The study received ethical approval from the St George’s University of London Research Ethics Committee (Ref 2022.0073).

### 2.2. Participants

Purposive sampling was used to capture information‐rich data from a population of PwP who had taken part in online exercise groups. Inclusion and exclusion criteria are listed in Table 1. Participants were recruited through the Parkinson’s UK Charity Take Part hub from May to July 2022. Participants were informed of the study through an online advertisement, and interested participants were provided with study information and contact details for the lead researcher (JA), who then completed eligibility screening. Following confirmation of eligibility, a mutually convenient time was arranged for participant interviews. Informed consent was obtained in writing through participants returning signed consent forms and then confirmed verbally prior to each interview.

**Table TABLE 1:** Inclusion and exclusion criteria.

Inclusion	Exclusion
Diagnosis of idiopathic Parkinson’s disease	Diagnosis of nonidiopathic Parkinson’s disease, e.g., PSP, MSA or other Parkinson’s plus syndrome
Aged over 18 years	
Participation in a ‘live’ online exercise group within 12 months prior to the interview	
Able to access technology to participate in an online interview	
English speaker to conversational standard	

As the aim was to identify broad themes on a specific issue, and purposive sampling was expected to generate a relatively homogeneous study population, the *a priori* estimated sample size between 8 and 10 participants was expected to capture the diversity of experiences. An iterative process of data analysis, including thematic analysis utilising both semantic and latent coding, was used to reach conceptual depth [28]. Conceptual depth as described by Nelson [29] considers the importance of having range, complexity, subtlety, resonance and validity within data. This was agreed to have been reached through collaboration between researchers (JA, CA and YC).

### 2.3. Data Collection

An interview guide created by the lead researcher (JA) was piloted with one PPI group member, to check for clarity and acceptability. Their data were not used in the final sample reported in the manuscript. The interview guide contained impartial, open‐ended questions. Initial questions focused on topics not specific to the research question such as their experience of research and exercise to familiarise participants with the interviewer and style of questioning. Subsequent interview questions focused on experiences of online exercise groups, rationale for participation, accessibility and perceived value. Follow‐up and prompting questions were used to deepen and seek clarification on participants’ answers.

Interviews were conducted online via Microsoft Teams between June and August 2022. Interviews were carried out by researchers (JA, CA and YC) and video recorded. All participants were interviewed alone by one researcher. Field notes were taken by interviewers during the interview, and the interviews were transcribed during video capture by Microsoft Teams which were checked verbatim by researchers (JA, CA and YC). Participants were sent copies of their transcripts for confirmation of accuracy.

### 2.4. Reflexivity

All interviewers are physiotherapists working in the UK National Health Service specialising in a neurological setting and have experience of clinical research. Their familiarity with supporting patients virtually during the COVID‐19 pandemic facilitated participants to explore the meaning behind their experiences and facilitate richer engagement [30]. To counteract bias and establish credibility, individual transcripts were sent to each participant for verification.

### 2.5. Data Analysis

Transcripts were uploaded into NVivo 12 [31] and analysed using thematic analysis [32]. Transcripts were read repeatedly to maximise information gained and analysed by the lead researcher (JA). Themes were created deductively from theoretical and conceptual frameworks underpinning the study and inductively based on familiarisation with the data [33]. The deductive coding process was informed by the interpretivist phenomenological approach and frameworks pertinent to lived experiences of physical activity and exercise interventions such as self‐determination theory [34]; the Capabilities, Opportunities, Motivators—Behaviour (COM‐B) model [35]; and social cognitive theory [36]. These frameworks shaped initial expectations around study themes and supported a set of deductive codes, including accessibility, instructor knowledge, social interaction and support, and behaviour change. These codes were considered alongside inductive codes emerging from the data to develop a codebook (see supporting information). This ensured both theory‐driven and participant insights were captured during data analysis. A sample of transcripts was analysed by researchers (JA and YC) for discussion and corroboration of themes. Individual transcript themes were sent to participants for verification and comment to enhance rigour and ensure participants’ views had not been misrepresented. Themes and subthemes were further refined through an iterative process by team discussion, and any areas of disagreement were resolved through consensus to ensure validity [37].

## 3. Results

Five female and four male adults were recruited (mean age 69.5 years, range 63–78 years; mean disease duration 9.1 years, range 3–20 years). All participants described their ethnic status as White British. Interviews lasted 47–90 min. Twelve different classes were attended by the nine participants. Reported exercise types included PD Warrior, boxing, ballet, Pilates, yoga, functional exercise, multicomponent exercise and HIIT. Class length was between 30 and 90 min, and frequency of classes was between 1 and 3 times per week. Ten of the 12 classes involved a cost to participate costing from £4–£10 per hour. Classes were led by physiotherapists (*n* = 7), fitness instructors (unknown experience) (*n* = 1), fitness instructors with PD experience (*n* = 1) and ballet instructors (*n* = 3). Transcript analysis revealed three overarching themes: *‘Considerations of online exercise groups for people with Parkinson’s disease*’, ‘*Online exercise class qualities’* and ‘*Accessibility’* (Figure 1).

**Figure FIGURE 1:**
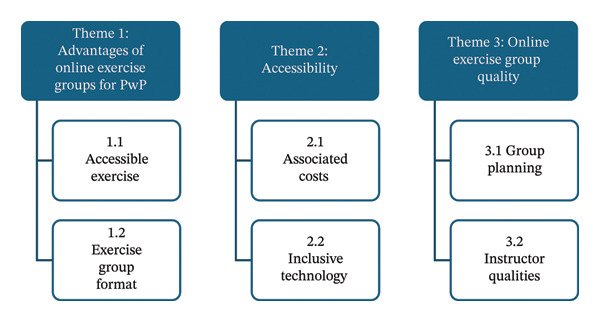
Overarching themes and subthemes.

### 3.1. Theme 1: Considerations of Online Exercise Groups for PwP

Participants valued online exercise groups to support their Parkinson’s symptoms through physical activity by improving accessibility to exercise. They felt online groups complemented in‐person groups as well as other support from health and care services.

#### 3.1.1. Convenience of Exercise for Physical Self‐Management

Participants felt that online groups increase accessibility to exercise for people with advancing disease, mobility difficulties or limited transport options by offering exercise at home.

“Pacing itself is important in Parkinson’s…I think I′d be easily put off going to a face‐to‐face [class] if the effort to get there was too much, you know, or “I better turn back today because the weather is bad”, you know, “because of the journey”. Whereas if it′s online it doesn’t matter what the weather is like really.” [Participant 03]

Participants recognised that when not able to access an in‐person class, such as during the COVID‐19 pandemic or with fluctuating symptoms, online exercise groups facilitated motivation and support to continue exercise habits to manage their Parkinson’s symptoms. This has continued since the pandemic.

“I felt I was missing the live classes and just trying to manage it on my own was not working enough. I didn’t feel I was sufficiently motivated or well organised to do what I needed to do.” [Participant 04]

#### 3.1.2. Preference of Delivery Format: Online vs. In‐Person

All participants had participated in various forms of in‐person exercise prior to attending the online classes. These were either in exercise groups or in team sports. All participants took part in online classes due to COVID‐19 social restrictions. Following the lifting of social restrictions, most participants reported preferring returning to in‐person classes when these become available. However, online groups were seen as a useful alternative for maintaining exercise routines. Participants recognised their value as a tool to support physical self‐management for PwP.

“I don’t ever think it’s going to be first choice. But it is very valuable if for whatever reason that the in‐person can’t be done, that might be because there’s something wrong with people or transportation or instruction to lockdown, whatever it might be it’s kind of like your backup plan.” [Participant 01]

Several participants expressed a desire for a hybrid approach, facilitating both in‐person and online opportunities to exercise. This could offer greater flexibility to access exercise.

“But at its worst it’s, there’s another possibility, so there is another tool if you will. It’s not just you gotta be there, it could be, well if you can’t get there…you get a mix of people phoning in, video linking in, being in person.” [Participant 01]

“Because at the moment I’ve got these three different exercises I do each week, one online, one in the swimming pool and one in person. And I like that sort of mix.” [Participant 02]

### 3.2. Theme 2: Online Exercise Class Qualities

Participants identified characteristics that are important for online exercise classes as well as the instructors.

#### 3.2.1. Class Aims, Structure and Content

Participants had experience with various online classes, specifically for PwP. They emphasised the importance of a choice of exercise options and clear class aims to match individual needs and preferences. This was important for PwP to manage their own symptoms.

“It’s always something that with any group that you’ll need to plan it very carefully, look at what their needs of the people are and how they’re going to interact. And if there’s somebody who’s needs, aren’t going to be met then that needs to be discussed with them and, you know, to look at all alternatives.” [Participant 05]

Participants advocated for exercise groups to suit the individual and changing presentation of Parkinson’s. There was a mixed opinion on how participants felt about being in a group of differing abilities and stages of PD.

“Actually, people who aren’t able to do a lot of the exercises in the more demanding classes I think offered perhaps a different one where people are functioning at much the same level. I think that it needs to remain open ended. You don’t want to get stuck.” [Participant 06]

Others saw benefit to having a more diverse group of participants with PD at different stages and the opportunities this can offer for peer support.

“No, I think it adds to it really. Because we’re in the same boat in some ways. And you know, we may not be like that now, but we may be one day.” [Participant 02]

Most participants preferred in‐person groups for their sense of community; however, online groups, including local groups, could still provide a social benefit, which is important for PwP.

“I mean, you can’t really beat face‐to‐face because at the end of the day, because there’s people there you can talk to and things like that and that’s a different ball game.” [Participant 03]

“I think there’s loads of people [with Parkinson’s] out there that would benefit if there was more available, particularly groups, you know, local groups that are still online. But where you’ve got the potential to make social contacts, possibly in the future.” [Participant 04]

For many participants, they felt that a social aspect within their online group was specifically important for PwP and providers should build opportunities for socialisation within their class.

“I think the ability to actually socialise a little bit, to not just turn up, watch the thing, turn it off and go back to your own little box. I think it does need that bit of banter and chatter at the beginning if you can, if there’s time.” [Participant 07]

“And I think with Parkinson’s you can get very down quite often. So, I think that that’s [a social element] quite important.” [Participant 08]

#### 3.2.2. Instructor Qualities

Participants commented on prerequisites instructors should have, most notably their need for knowledge of PD and how exercise approaches may differ for PwP, depending on their symptoms. They also acknowledged the new skills needed to deliver exercise to a group through a virtual medium.

“They need to fill me with confidence that they know what they are doing. They’re not going to put me in any danger with any exercise. And that what we are doing is actually going to be helpful and that they understand why and how.” [Participant 05]

Some felt that instructors should be qualified physiotherapists with specific knowledge and experience of PD.

“A basic physiotherapist, you need to be a qualified physio really to start off. Then having some speciality in a particular form such as Parkinson’s or neurological to be able to do the things they do in the manner that they do it.” [Participant 09]

### 3.3. Theme 3: Accessibility

Participants highlighted challenges in making online exercise groups inclusive for all PwP. They focused on barriers like cost, digital illiteracy and digital poverty.

#### 3.3.1. Associated Costs

Participants noted that PwP often experience financial insecurity due to advancing disease, reducing their ability to work. Associated costs of online exercise classes, such as attendance fees and purchasing of exercise equipment, could be a barrier to participation. While most participants paid to attend classes and were willing to cover some costs, reduced income made affordability a key concern.

“It’s worth the money, but it is quite a chunk out of my income, which is far less than when I was working, you know. I don’t have the same budget now.” [Participant 03]

“I mean I’ve talked to people with Parkinson’s who say “Well, I want to do 3 classes a week, but they’re £10 each, that’s £30 a week and I can’t really afford it”. It’s as simple as that. You know, people are on benefits or very low pay. They’ve got to make choices. So yeah, it’s tricky.” [Participant 04]

Some participants highlighted that online exercise class providers were working with national charities, such as Parkinson’s UK, to offer financial support for those on low incomes to help access classes.

“They [exercise provider] are also trying to improve their services and have opportunity for people who are less fortunate than us to be able to have access to the classes through a bursary fund which they are wishing to set up with support from Parkinson’s UK.” [Participant 09]

#### 3.3.2. Inclusive Technology

Participants shared that challenges with technological illiteracy could be more prevalent in PwP or older adults, and this could be a barrier to participating in online exercise classes.

“I think, bearing in mind a lot of people with Parkinson’s, like my sort of age group, it doesn’t come naturally to us.” [Participant 02]

Participants reported experiencing technological issues related to connectivity and interaction with the platform interface. All participants felt that they could overcome these challenges.

“I suppose early on, there was hardly a week went by when somebody was having problems getting online and I’ve had an occasional time when I couldn’t get the sound to work or I couldn’t get a signal. Or it was my fault. Yeah, so, but I mean you get better with practice and I don’t think that’s much of an issue now actually.” [Participant 02].

There were concerns that digital poverty could exclude PwP from accessing and engaging in online exercise groups. Participants felt it was important to support those with reduced technological literacy or those without appropriate IT equipment.

“…obviously making sure that people have got the right equipment to do it with and you know, maybe there’s a way of finding, some sort of charitable way people could be helped…” [Participant 07]

“Perhaps put in place things for people who are new to use the technology that can help them…some written instructions that are very simple.” [Participant 05]

Participants experienced challenges with awareness of Parkinson‐specific online exercise groups. This was identified as a barrier to accessing groups. They felt clearer signposting and advertising were needed. This could be supported by providers and healthcare providers working more closely together.

“I tried to find a local class and couldn’t. I mentioned this to the Parkinson’s nurse who came back to me with the information about this group.” [Participant 05]

“That’s where early intervention and signposting needs to be done at doctors’ practices let alone consultant’s specialists in Parkinson’s.” [Participant 09]

## 4. Discussion

### 4.1. Overall Summary

The aim of this study was to explore PwP’s views and experiences of online exercise groups. The recruitment strategy captured experiences of a variety of online exercise groups across the UK. Unlike previous research focused on individual programs, this study examined a range of exercise types, instructors and class formats to provide a broad understanding. PwP all considered online exercise groups to have value in supporting physical self‐management but lack the social connection that many PwP seek. Participants acknowledged the benefits of online groups in reducing some of the barriers to accessing in‐person groups they and many PwP experience: the many challenges of physical restrictions including varying mobility, poor balance and fatigue and environmental barriers such as a lack of access to transport or the location of the nearest group. This, in turn, supported physical self‐management. Challenges remain in ensuring that groups are delivered by knowledgeable instructors and exercise delivery is tailored to address both Parkinson’s symptoms and individual needs. Accessibility did not seem to be a significant barrier to PwP, although some challenges with familiarisation with technology were experienced.

### 4.2. Context

#### 4.2.1. Considerations of Online Exercise Groups for PwP

Participants described a stronger sense of community within in‐person groups, which they felt was often missing in online formats, in keeping with previous studies [23, 38]. However, participants desired continued access to online exercise groups to support the management of Parkinson’s symptoms. This aligns with recent studies, demonstrating online groups improved access to exercise for people with unpredictable symptoms, advancing disease and mobility or transport limitations [20, 23, 39]. The duality of PwP wanting both online and in‐person groups underscores the need for flexible, hybrid models. This has been alluded to elsewhere [40, 41]. Hybrid models could include classes alternating between in‐person and online delivery, the option to attend a class in‐person and/or remotely, or an exercise class transitioning from in‐person classes to remote classes. Hybrid exercise classes are seen as feasible, safe and demonstrate promising effects on physical and cognitive domains in PwP [42], long COVID [43] and cardiovascular disease [44]. Hybrid approaches may help increase equity of access to physical activity and provide the sense of community many PwP seek. Further definitive clinical trials are needed to explore the optimal ways hybrid formats (combining both in‐person and online delivery) can be applied to deliver group exercise interventions for PwP. This will help to identify the most appropriate format for different intervention types or intended outcomes.

#### 4.2.2. Online Exercise Class Qualities

Participants in this study highlighted the importance of understanding how an online exercise class could help them manage their Parkinson’s symptoms. Many expressed that knowing the purpose and expected benefits of a class was key to choosing one that aligned with their personal goals to improve their Parkinson’s symptoms, e.g., balance, walking or coordination. Relevance of an exercise class and tailored support can aid motivation and adherence to exercise [45–47]. Clear class aims and appropriate exercise delivery should be considered by those designing online exercise groups for PwP. Participants in this study perceived that this sense of relevance was closely tied to their motivation and long‐term adherence. This is important for physical self‐management in PD. We argue for greater working between exercise providers and PwP to codesign online exercise groups that meet the specific needs of PwP. Previous studies have found e‐Health interventions can aid motivation and adherence to exercise [48], as well as support behaviour change to self‐manage symptoms in PD [49] and Type 2 diabetes mellitus [50]. It needs to be ascertained if e‐Health physical activity interventions can support physical self‐management in PD.

Supporting physical self‐management in PwP requires exercise providers to have sufficient knowledge and skills in online exercise delivery and behaviour change [38]. In this study, all participants felt that instructors need to have knowledge of exercise and PD. Some participants felt strongly that instructors should be qualified physiotherapists. This relates to trust in their instructor to minimise potential harm from exercise and have knowledge of exercise for managing PD symptoms, as well as skill, using an online platform to deliver exercise, observe participants and provide tailored feedback. It is known that PwP are more willing to exercise if supervised by qualified professionals [46]. Physiotherapists with expertise in PD and exercise can deliver cost‐effective care for PwP [51], but relying on classes run by physiotherapists alone presents pragmatic problems with supply and demand. Given participants’ concerns about instructor qualifications, future research is needed to identify the skills, attributes and expertise needed to safely and effectively deliver online exercise groups for PwP.

#### 4.2.3. Accessibility

Previous literature has highlighted high costs as a deterrent to participation in exercise [46]. Participants in our study described similar thoughts with high class costs influencing the choice and number of classes participants could attend. PwP are known to be financially worse‐off compared to the general population [52]. Online groups could aid exercise participation by reducing travel costs associated with in‐person exercise. However, costs associated with purchasing equipment, including smartphones and laptops, and poor internet connectivity could further reduce access for PwP from lower socioeconomic groups [53, 54]. Within our study, participants identified collaborations between charities and online exercise group providers to offer subsidised rates. We argue that further collaboration between exercise providers, charities and local councils could support equitable access to online exercise for PwP from lower socioeconomic groups.

Online groups risk inequity of access to those without computer literacy, often prevalent in older adults and those with cognitive impairment, common characteristics of PD [55]. Consideration of technical support for participants prior to starting online groups through one‐to‐one sessions and/or provision of written information could improve equity of access [41, 48]. Poor advertising of online exercise groups for PwP was a barrier to participation, with participants relying on family or healthcare professionals for signposting. Greater opportunities for collaboration between online exercise group providers and the healthcare and charitable services that PwP use could enhance awareness of online groups.

### 4.3. Strengths and Limitations

The sample size was relatively small, but conceptual depth was achieved. The sampling strategy allowed for recruitment of males and females with a range of years since diagnosis, allowing us to consider findings in the context of those in early, mid and late‐stage PD. All participants described themselves as White British; therefore, the findings may not represent other ethnic groups. Further research is needed to ascertain the views of under‐represented populations, including those from BAME and lower socioeconomic backgrounds. Additionally, the perspectives of exercise professionals delivering online classes warrant investigation. The study only sought to explore experiences of those who had participated in online exercise groups and did not capture the views of PwP who did not wish or were unable to participate in online exercise classes.

## 5. Conclusions

Online exercise groups may play an important role in future Parkinson’s management, but they lack the social engagement that many PwP seek. The integration of both in‐person and online exercise groups in a hybrid model may offer greater opportunities for PwP to manage their symptoms through sustained physical activity habits. PwP choose online exercise groups that are specific to their individual needs and goals and delivered by instructors with PD‐specific knowledge. Collaboration between providers and PwP to codesign suitable interventions should be considered. Online exercise groups can offer greater access and adherence to exercise but may perpetuate inequalities for PwP with limited access to equipment and the internet. Future investigation should focus on how collaboration between providers, health and charitable services, and PwP could improve awareness of online exercise groups.

## Author Contributions

Mr James Alexander (primary investigator) contributed to study design, data collection, data analysis and primary author.

Dr Caroline Appel contributed to data collection, data analysis and review of the manuscript.

Ms Yiota Constantinou contributed to data collection, data analysis and review of the manuscript.

Professor Anette Schrag provided support and guidance with study protocol development and review of the manuscript.

## Funding

James Alexander received support and funding from Health Education England and the National Institute for Health Research. This study was undertaken during his HEE/NIHR ICA programme predoctoral clinical and academic fellowship (NIHR301892).

## Disclosure

The study was presented as a poster at the World Parkinson’s Congress in July 2023. A subsection of the findings was presented as a poster at the Chartered Society of Physiotherapy conference in November 2023.

## Conflicts of Interest

The authors declare no conflicts of interest.

## Supporting Information

Additional supporting information can be found online in the Supporting Information section.

## Supporting information


**Supporting Information 1** Interview topic guide. The included interview topic guide was used to undertake the semistructured interviews with all participants in this study.

**Supporting Information 2** Codebook. A set of deductive codes were considered alongside inductive codes emerging from the data to develop a coding framework. This ensured both theory‐driven and participant insights were captured during data analysis.

## Data Availability

The data that support the findings of this study are available from the corresponding author upon reasonable request.
