# Supplementary material for: Views and Experiences of Online Exercise Groups for People With Parkinson’s Disease: A Qualitative Study
Source: Parkinsons Dis. 2026 Jan 28;2026:9816558. doi: 10.1155/padi/9816558 (PMC12848610; doi:10.1155/padi/9816558)
Supplement: Supplementary file 1 — Supporting Information 1 Interview topic guide. The included interview topic guide was used to undertake the semistructured interviews with all participants in this study. [file PADI-2026-9816558-s001.docx]

Exploring the experiences people with Parkinson’s Disease have with online exercise groups.

Interview guide

Aim

What are the experiences of PwP who have utilised online exercise groups in managing their condition?

Secondary Aim(s)/Objectives

To establish if PwP find online exercise groups accessible, of value and effective in supporting them to self-manage their condition through increasing their physical activity.

- To explore individual rationale for participating in online exercise.
- To explore individual experiences of the accessibility of having attended online exercise groups.
- To explore individual experiences of the health-related value (including perceived impact and effectiveness) for themselves of having attended online exercise groups.
- To explore the perceived impact and effectiveness of setup and delivery of online exercise groups.
- To explore any differences in experiences between those at different stages of PD (e.g., early, mid or late-stage PD).

Before the admitting the participant into the meeting:

1. Check your microphone and camera are switched on and working
2. Click on the 3 dots (more) option on the top bar and select “start recording”
3. Click on the 3 dots (more) option on the top bar and select “start transcription”
4. Admit the participant into the meeting and support them as needed to turn on their camera and microphone. If they need assistance click on the people tab on the top bar which brings up the participants in the meeting. When you hover the cursor over their name 3 dots appear. Click on these and you will have the ability to disable mic and disable camera. You may need to add them as an attendee first (rather than a guest) to have the rights to do this.

| **Theme/objective** | **Questions** | **Prompts** |
| --- | --- | --- |
| **Introduction** | Introduce self  *“This research is being conducted to get to know the views of people with PD who have participated in “live” online exercise groups. I am conducting this research as a Senior Lecturer in Physiotherapy at St George’s University of London/Physiotherapist working with people with PD in the NHS. I am especially interested in the experiences and perspectives of people with PD who have participated in online exercise groups during the Covid-19 pandemic. I am interviewing people from all over the UK with help from Parkinson’s UK. The questions I would like to ask you relate to the topics of online exercise groups and PD. It is hoped the data we collect can help with development of rehabilitation services for people with PD. Everything you tell me will only be used for this research project and will not be shared with anyone outside the research team. Also, your name will not be used, to make sure that no one can identify you with any answers. Your data and that of others will be analysed together to see what similar themes and topics arise. This will be then written for journal publication. The findings will also be fed back to you by a presentation through Parkinson’s UK. The interview will be audio and video recorded to help with us with transcribing your answers. You have already signed a consent form but first I would like to check that you are happy to continue with the interview. Do you have any questions before we begin?”*  Please check that you are recording (video if consented) and that transcription is taking place |  |
| **General questions** | | |
|  | Q1 Have you ever taken part in research? If so, what are your experiences? | What made them interested in this study? |
|  | Q2 Can you tell me a little about your Parkinson’s disease? | Time since diagnosis  Main symptoms |
|  | Q3 How have you found the transition to online communication during the pandemic? | Were you familiar with zoom or teams before?  How did you stay in touch with family? Were medical appointments shifted online? |
| **Opening questions**  **“I would like to ask some questions on exercise and Parkinson’s disease now”** | | |
| Individual rationale for participating in online exercise | Q4 What are your thoughts about the role of exercise in general for managing PD? | What reasons do you think people have for exercising?  What reasons do you have for exercising? |
|  | Q5 What do you think about the role of exercise groups for managing PD? | For PWP, or specifically for you and your symptoms? |
|  | Q6 During the last year, other than the online live groups, have you participated in any other exercise classes for you PD? | Were these face to face or pre-recorded, individual sessions?  If not, why? |
| **“I’m now going to focus on your experiences of “live” online groups”** | | |
| Individual rationale for participating in online exercise | Q7 What were your reasons for participating in an online exercise group(s)? | Why this group/these groups?  What were you wanting to get out of the group? |
| Effectiveness of setup and delivery of online exercise groups | Q8 Tell me about the setup and structure of the online exercise group(s) you have participated in? | Tell me about the people involved in the group.  What length of time did the group run for? Was it free or a cost involved?  What was particularly good or bad about the setup and structure of the group? |
|  | Q9 Can you tell me what you would do during the class? | Can you tell me about the exercise component?  Did the group have any components other than exercise e.g. education, peer-support or social? |
|  | Q10 What do you think should be included and not included in an online exercise group? | Why are these important for PWP?  Why are these important for you? |
| Experiences of the health-related value | Q11 Tell me about how the group impacted on your health and well-being? | Did you notice any changes to your PD symptoms, mood or well-being?  How do you think these changes came about? |
| Experiences of the accessibility | Q12 Tell me about your experiences of accessing the online group(s). | What equipment did you need (tech or exercise equipment)?  Did you experience any accessibility difficulties that affected your ability to engage with the class? |
|  | Q13 What worked well and what should improve in terms of accessibility of online exercise groups? | What recommendations would you give to someone setting up an online class?  What recommendations would you give to someone wanting to participate in an online class? |
| Experiences of the health-related value | Q14 What are your thoughts on the value of online exercise groups? | How do online and face-to-face groups compare? |
| **Closing questions** | | |
| Value and effective in supporting them to self-manage their condition | Q15 How do you feel online exercise groups could support people with PD to manage their condition? |  |
|  | Q16 Do you see online exercise groups playing a role in your own self-management of your PD? | How so? |
| Thank you for taking the time to tell me a little bit about your experiences of online exercise groups. Is there anything else you think I should know? | | |
