# Supplementary material for: Views and Experiences of Online Exercise Groups for People With Parkinson’s Disease: A Qualitative Study
Source: Parkinsons Dis. 2026 Jan 28;2026:9816558. doi: 10.1155/padi/9816558 (PMC12848610; doi:10.1155/padi/9816558)
Supplement: Supplementary file 2 — Supporting Information 2 Codebook. A set of deductive codes were considered alongside inductive codes emerging from the data to develop a coding framework. This ensured both theory‐driven and participant insights were captured during data analysis. [file PADI-2026-9816558-s002.docx]

Codebook Template

This codebook provides a structured framework for qualitative analysis.

| **Code** | **Underpinning framework** | **Definition** | **Inclusion Criteria** | **Exclusion Criteria** | **Example Quote** |
| --- | --- | --- | --- | --- | --- |
| Convenience of exercise | COM-B model: opportunities | References to ease of accessing exercise online | Mentions of reduced travel, flexibility, or ability to exercise at home | General comments about exercise benefits without reference to convenience | “Whereas if it's online it doesn’t matter what the weather is like really.” |
| Preference for delivery format | COM-B model: motivators | Statements comparing online vs in-person classes | Mentions of hybrid models or backup plans | Comments unrelated to delivery format | “I don't ever think it's going to be first choice. But it is very valuable if for whatever reason the in-person can't be done.” |
| Social interaction | Self-Determination Theory (relatedness) | Importance of social engagement in classes | Mentions of banter, community, or loneliness | General comments about exercise benefits without social context | “I think the ability to actually socialise a little bit…not just turn up, watch the thing, turn it off.” |
| Instructor qualities | Social Cognitive Theory (observational learning) | Attributes or qualifications expected of instructors | Mentions of PD-specific knowledge, physiotherapy qualifications | Comments about class content rather than instructor | “They need to fill me with confidence that they know what they are doing.” |
| Cost barriers | Social cognitive theory (environmental factors) | Financial challenges in accessing classes | Mentions of affordability, bursary funds | Comments about technology or awareness | “I want to do 3 classes a week, but they’re £10 each, that's £30 a week and I can't really afford it.” |
| Technology barriers | COM-B model: Capabilities | Challenges with digital literacy or equipment | Mentions of connectivity, instructions, or digital poverty | General comments about exercise benefits | “I think, bearing in mind a lot of people with Parkinson's, like my sort of age group, it doesn't come naturally to us.” |
